# Eosinophils Infiltration in Esophageal Muscularis Propria Induces Achalasia-like Esophageal Motility Disorder in Mice

**DOI:** 10.3390/biom12121865

**Published:** 2022-12-13

**Authors:** Wei Zhao, Bin Wang, Lili Zhang, Hong Jin

**Affiliations:** Department of Gastroenterology and Hepatology, Tianjin Medical University General Hospital, Tianjin 300052, China

**Keywords:** achalasia, eosinophil, mice

## Abstract

Eosinophil infiltration in esophageal muscularis propria is common in achalasia (AC). This study aims to evaluate the effect of eosinophil infiltration in muscularis propria of the esophagus on esophageal motility in mice. A mouse model with eosinophil infiltration in the esophageal muscle layer was established by long term Ovalbumin (OVA) exposure. The histopathology features of esophageal muscularis propria as well as parameters of esophageal motility, such as lower esophageal sphincter pressure (LESP) and esophageal emptying, were compared between model and control group. In addition, the histopathology and motility of esophagus at each time point in the model group were compared. The esophageal motor function severely deteriorated in the model group, mimicking the abnormal esophageal motility of AC, with more eosinophils and fewer SOX-10-IR cells in esophageal muscularis propria in the model group, compared with control. With the prolongation of OVA treatment, esophageal motility disorder was aggravated, accompanied by increased eosinophils in the the muscle layer of esophagus and decreased SOX-10-IR cells in the model group. In addition, the eosinophil count was negatively correlated with SOX-10-IR cells. Long-term exposure to OVA assisted by alum may induce eosinophil infiltration in esophageal muscularis propria, reduced SOX-10-IR cells and abnormal esophageal motility, which simulates the functional and histopathological features of some AC patients. This suggests that eosinophil infiltration in esophageal muscularis propria may play a role in the pathogenesis of a subgroup of AC.

## 1. Introduction

Idiopathic Achalasia (AC) is a rare motility disorder of the esophagus, manifested by dysphagia, regurgitation of undigested food, respiratory symptoms, chest pain and weight loss [1]. The pathogenesis of this disorder remains unknown, and accordingly all current treatment modalities, such as laparoscopic Heller’s myotomy, pneumatic dilation, or per-oral endoscopic myotomy (POEM) are aimed at symptom control as opposed to disease cure. Although the short-term effect of these treatments is usually satisfactory, the long-term effect is not ideal, which may be due to the progression of the disease [2,3]. This prompts in-depth study of the pathogenesis of AC.

Immune-mediated ganglionitis may induce esophageal myenteric plexus injury in AC, with various inflammatory cells, including eosinophil, participating in this process [4,5,6]. We also found eosinophil infiltration in the muscle layer of the esophagus and its positive correlation with ganglionitis and myenteric plelux damage in AC patients [7], as substances released by eosinophils, such as eosinophil cationic protein (ECP) and eosinophil-derived neurotoxins (EDN), can damage nerves [8], and eosinophil infiltration in the esophageal muscle before POEM is associated with a poor prognosis of treatment (Z = 3.50, *p* = 0.030) [7]. Although there are different opinions [9], due to the complex pathogenesis of AC, and the small sample size of existing studies, we still speculate that eosinophil infiltration in the esophageal smooth muscle may be associated with the pathogenesis of some AC patients. However, the lack of an animal model with eosinophil infiltration in the esophageal muscularis propria is a major obstacle for further studies.

Since AC-like dysmotility can also be seen in some eosinophilic esophagitis (EoE) patients [10], using an animal model of EoE as a surrogate is a potential solution. Among various methods to establish an animal model of EoE [11,12,13,14], the respiratory tract antigen, such as ovalbumin (OVA), exposure is simple, low cost and easy to control experimental conditions [15]. However, to the best of our knowledge, the depth of the eosinophil infiltration is mainly in squamous-epithelial, lamina propria and submucosa of esophagus in the mouse models listed above [11,14,15], which is not consistent with previous pathological finding in AC. We assume that in sensitized mice, compared with traditional methods, longer respiratory antigen exposure can induce eosinophil infiltration in muscularis propria, damage of myenteric plexus and AC-like dismotility of the esophagus.

In this study, we aim to test this hypothesis: long-term respiratory exposure of sensitized mice to OVA can promote a large number of eosinophils to infiltrate the smooth muscle of the esophagus, accompanied by decreased myenteric nerves and AC-like esophageal dismotility. In addition, the relationship between antigen exposure time and the histopathology of esophageal muscularis propria, as well as abnormal esophageal motility, was studied.

## 2. Material and Methods

### 2.1. Animals and Study Protocol

The experiments were approved by the Animal Welfare and Ethical Committee of Tianjin Medical University General Hospital, Tianjin, China (No. TMUGH2020-12-0027). All animal experiments were performed in accordance with the SOP Operating Procedures for Laboratory Animal Center, Tianjin Medical University.

Four to six weeks old male BALB/c mice (Animal License No. SCXK (Beijing) 2016-0006) were provided by Beijing Victoria Laboratory Animal Technology Co., Ltd, Beijing, China. They were housed in a temperature-controlled facility with a 12-h light/dark cycle and were given free access to diet and water. As shown in the flow chart, one week after adaptive feeding, mice were subdivided into two experimental groups: control (CN, n = 20) and esophagitis infiltration group (OVA sensitization as model group, n = 40). The body weight, lower esophageal sphincter pressure (LESP), esophageal width and esophageal emptying were compared at every time point (baseline (D0), D21, D28 and endpoint (D35)) between groups, respectively. Pathology of esophageal wall at baseline and endpoint were also compared between groups. Furthermore, the parameters of esophageal motility and pathology at each time point were compared in the model group.

**Figure 1:**
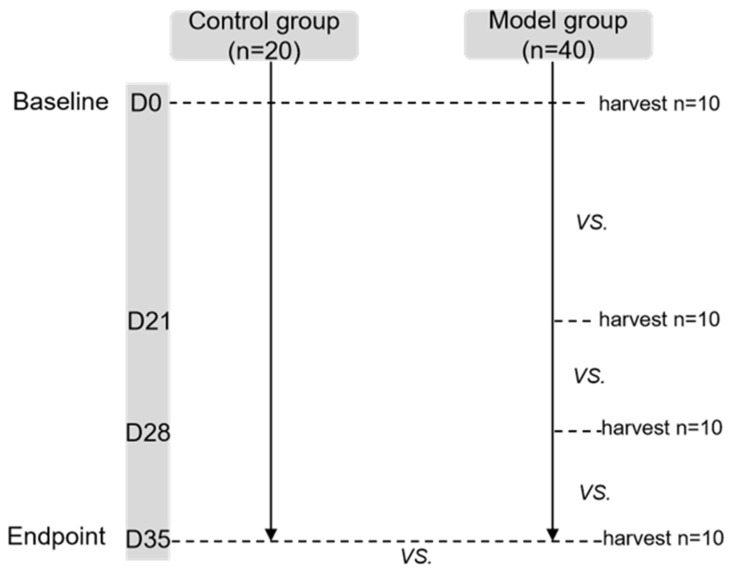
Flow chart of the study. In the model group, the LESP, esophageal emptying and histological results at every viewpoint (D21, D28, D35) were compared with baseline (D0). At the endpoint of the study (D35), the low esophageal sphincter (LES) pressure LESP, esophageal emptying and histology of mice in the control group and model group were compared. In each viewpoint, the LESP was first programmed, and followed by the esophageal emptying test. Finally, 10 mice were selected randomly and sacrificed for esophageal histology at every time point in model group, and at D0 and D35 respectively in the control group. LESP: lower esophageal sphincter pressure.

In the model group, 10 mice were randomly selected at each time point for esophageal pathological examination. In the control group, 10 mice were randomly selected for the same examination at baseline and endpoint. The lottery method was used for randomization (Figure 1).

### 2.2. Mouse Model with Eosinophil Infiltration in Esophageal Muscularis Propria

A mouse model with eosinophilic infiltration in esophagus was established as previously described with some modification (prolongation of respiratory antigen exposure from 28 to 35 days) [15]. In brief, mice were lightly anesthetized with 3% iso,-flurane inhalation (methoxy-fluorane; Schering-Plough Animal Health, Union, NJ, USA), and sensitized by intraperitoneal injection of 50 μg of ovalbumin (OVA, Grade V, Sigma, St. Louis, MO, USA) and 1 mg alum (Sigma, St. Louis, MO, USA) in PBS (50 μg/1.0 mg/0.5 mL) on two occasions separated by 14 days. From the 15th day, under the condition of anesthesia induced by inhalation of 3% iso-flurane (methoxy-fluorane; Schering-Plough Animal Health, Union, NJ, USA), mice were intra-nasally injected with 150 μg OVA (50 μL) (Grade V, Sigma, St. Louis, MO, USA) using a micropipette with the mouse held in supine position. The above operation was performed three times a week for three weeks. Except for the last challenge, mice were given normal drinking water and diet 1 h after each operation while mice in the control group (CN) were sensitized and challenged with the same volume of PBS solution as model group.

**Figure 2:**
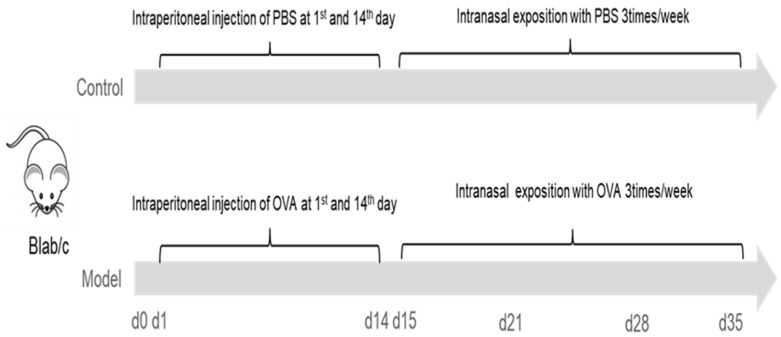
Eosinophil infiltration induction protocol. In the model group, at D1 and D14, mice were sensitized by intraperitoneal injection of 50 μg OVA and 1 mg alum in PBS (50 μg/1.0 mg/0.5 mL), and further challenged with 150 μg OVA (50 μL) intra-nasally, under the condition of anesthesia, 3 times per week for 3 weeks. Mice in the control group were sensitized and challenged with the same volume of PBS solution as the model group. OVA: ovalbumin.

In the model group, mice were sacrificed (by cervical dislocation) randomly (using lottery method) at base line and 18–20 h after the nasal attack on D21, D28 and D35, respectively. Mice in control group were also harvested randomly at base line and D35 (Figure 2).

### 2.3. Esophageal Manometry

High-resolution manometry of the esophagus, which is widely carried out in humans, cannot be applied to small animals. Therefore, integrated relaxation pressure (IRP) [16] was generally replaced by LESP in mouse experiments.

LESP was measured 18 h after fasting. In order to avoid asphyxia, we used a single-channel pressure gauge and kept mice at a position of 60 degrees during the experiment. Intraluminal esophageal manometry was performed using a specially designed micro-sized catheter with one micro-transducer (MMS-G-84300, SAR-MED. S.R.L, Iglesias, Italia). Mice were lightly anesthetized with 3% isoflurane inhalation for induction (methoxy-fluorane; Schering-Plough Animal Health, Union, NJ, USA) before intubation and then anesthetized at concentrations of 1.5% for maintenance during the examination. The mano-metric tracings were recorded by a water perfusion pressure measurement system (MMS, Rotterdam, The Netherlands). Manometry was carried out by a stationary pull-through method with the catheter placed trans-orally into the stomach of the spontaneously breathing mouse. The water infusion rate was 0.15 mL/min. The mean LESP for each mouse was calculated by measuring the pressure of the LES in resting state for 1 min three times (the average of the sum of three LESP); for each time, the tube was relocated with the tube rotated 60 degrees clock wise. The evaluation of the tracings was blinded and assessed by a senior gastroenterologist. The duration of esophageal manometry for each mouse was about 4–5 min.

### 2.4. Esophageal Emptying and Esophageal Radiography

**Figure 3:**
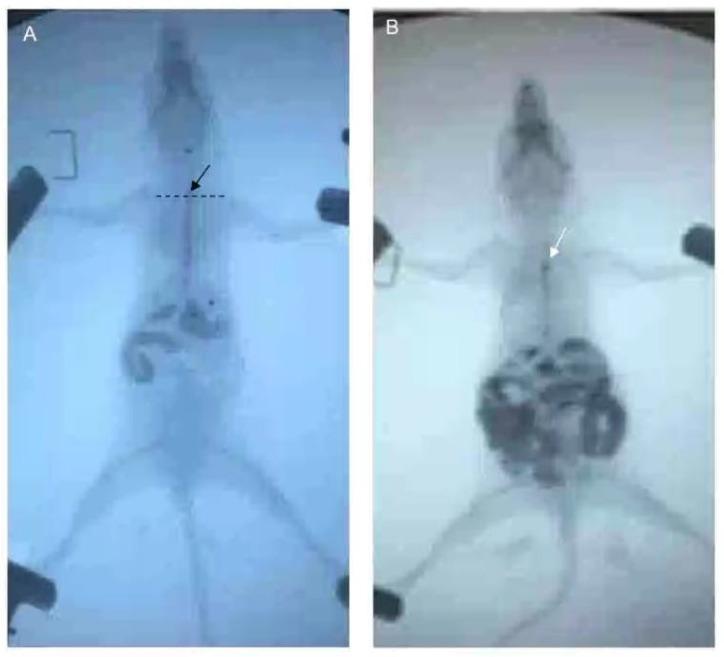
Esophageal emptying test in mouse. (**A**) Contour of the esophagus with 0.1 mL iohexol as contrast agent. The release position of the X-ray opaque marker was at the level of sternum angle shown with black arrow. (**B**) The movement of the X-ray opaque marker through esophagus. White arrow refers to the marker.

After fasting and water deprivation for 18 h, the mice were kept in supine position and lightly anesthetized with 3% iso,-flurane inhalation for induction and 1.5% for maintenance (methoxy-fluorane; Schering-Plough Animal Health, Union, NJ, USA). After that, a 12-gauge blunt-ended stainless steel animal feeding needle (Beijing Jingkaida Instrument Co., Ltd., Beijing, China) was inserted trans-orally to the upper part of esophagus at the level of sternum angle and 0.1 mL iohexol (320 mg/mL, Beijing Beilu Pharmaceutical Co., Ltd., Beijing, China) was injected into the stomach through the esophagus. The width of the widest esophagus under X-ray fluoroscopy is defined as the width of the esophagus. Then, another gavage needle (12-gauge, Beijing Jingkaida Instrument Co., Ltd., Beijing, China) with modified tip to maintain an X-ray opaque marker (stainless steel ball with 1 mm in diameter, Yuncheng County Kangda Steel Ball Co., Ltd., Yuncheng, China) was inserted trans-orally to the upper part of esophagus (at the level of sternum angle). Then, 0.1 mL of air was rapidly injected into the stomach via the gavage needle, driving the release of the marker to the esophagus at the sternal angle. The contour of esophageal cavity, the location of the marker and the whole process of its passing through the esophagus were monitored and recorded by X-ray fluoroscopy (SiemensAXIOMIconosR200, Siemens, Co. Ltd., Berlin, Germany). The time required for the marker to reach or pass through the cardia was recorded as esophageal transit time and cardia passing time, respectively. X-ray fluoroscopy was performed by an experienced radiologist. The duration of esophageal emptying test for each mouse was about 15–30 min. The data was assessed by two senior gastroenterologists who were blind to grouping. Any disagreement was settled by consensus (Figure 3).

### 2.5. Tissue Preparation and Eosinophil Count

Pathological analysis of LES was performed. The esophageal tissue at the site of LES was cut quickly and fixed in 10% formalin (Sigma, Buchs, Switzerland) immediately after harvest, washed twice in 70% ethanol (Merck Millipore, Darmstadt, Germany), stored in ethanol until embedding in paraffin (VWR, Tissue Tek VIP 5JR; Sysmex, Yverdon, Switzerland), and then cut into 5 μm sections (Zeiss Hyrax KS34; Histocom, Zug, Switzerland). Sections were processed for hematoxylin-eosin (HE) staining and immunohistochemistry.

The sections were stained with hematoxylin and eosin (H&E) (Beijing Zhongshan Jinqiao Biotechnology Company, China) and observed under a light microscope (BX51, OLUMPUS, Japan) by two experienced examiners. Eosinophilic density was estimated as the mean number of cells in 10 microscopic fields at 400 magnifications.

### 2.6. Immuno-Histochemical Staining

Immuno-histochemical staining for major basic protein (MBP), eosinophil cationic protein (ECP), eosinophil-derived neurotoxins (EDN) and SOX-10 were performed to identify activated eosinophils and myenteric plexus of the esophagus, respectively.

Immuno-histochemical staining was performed as described previously [17]. Briefly, paraffin sections (n = 10/group) were successively placed into xylene, high-to-low concentration alcohol and then repaired antigen. Sections were incubated with goat serum for 15 min. The following primary antibodies were used: Mouse monoclonal antibody Sox10 (1:500, Cat# ab155279, Abcam, Waltham, MA, USA), Mouse monoclonal antibody MBP (1:500, Cat# ab11159, Abcam, Waltham, MA, USA), Rabbit monoclonal antibody Ribonuclease3/ECP (1:500, Cat# ab207479, Abcam, Waltham, MA, USA), Rabbit polyclonal antibody EDN (1:500, Cat# ab238562, Abcam, Waltham, MA, USA). The primary antibodies were added to the sections and incubated at 37 °C for 60 min. Secondary antibodies were applied at room temperature for 20 min. Diaminobenzidine (DAB) was applied for 5–10 min. Hematoxylin staining solution was applied for 20 s. The sections were dehydrated and transparently sealed with neutral gum. Negative controls were prepared by omission of the primary antibodies. Positive controls were carried out according to the recommendation of the manufacturer of the antibodies. Images were acquired under an optical microscope (BX51, OLUMPUS, Tokyo, Japan).

Pathological examination and immunohistochemistry of the esophageal tissues was performed by two pathologists and a physician who were blinded to grouping. Any disagreement in their opinions was settled by consensus.

### 2.7. Immunohistochemical Evaluation

Semi-quantitative analysis of the immuno-histological staining was carried out in a blinded fashion by light-microscopy using a previously published protocol [18]. In brief, the impairments of myenteric neurons of the esophagus and the staining of MBP, ECP and EDN were evaluated by immuno-histochemical score (IHS). The IHS is calculated by multiplying an estimate of quantity score (1–10% scored as 1, 11–50% as 2, 51–80% as 3, and 81–100% as 4) with an estimate of the staining intensity score (0 = negative; 1 = weak; 2 = moderate, and 3 = strong). The IHS range is from 0 to 12. An IHS score of 9–12 was considered as strong immune-reactivity, 5–8-as moderate, 1–4 as weak, l and 0 as negative.

### 2.8. Statistical Analysis

Body weight, parameters of esophageal motility and esophageal emptying as well as eosinophil count and IHS were expressed as the mean ± SD. Normality was checked with Kolmogorov-Smirnov test. Comparison between groups and within model group were analyzed using unpaired *t*-tests or the Mann-Whitney U-Test as appropriate. The correlation between eosinophil count and SOX-10-IR cells of esophagus was analyzed by Spearman rank correlation. The threshold significance level was set at *p* < 0.05 for all tests. Statistical analysis was performed using SPSS software version 21 (IBM, New York, NY, USA).

## 3. Results

### 3.1. General Condition and Body Weight of Mice

**Figure 4:**
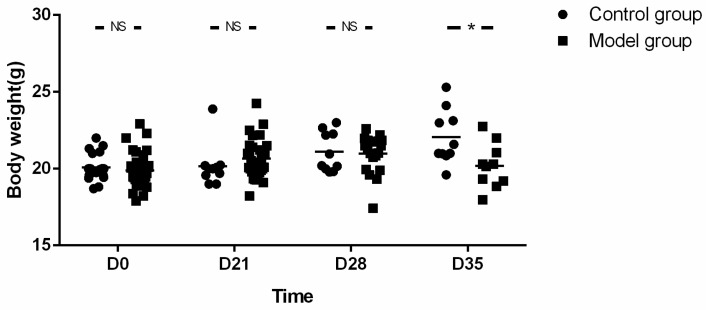
Body weight of mice. With the prolongation of OVA exposure time, body weight of mice in the model group was lower than in control group at the end of the experiment. Unpaired *t*-tests was used in comparison between groups and among different viewpoints in the model group. LESP: lower esophageal sphincter pressure. * *p* < 0.05, NS—not significant.

The mice in the model group showed significant reduction in the amount of food intake from the D21 and showed symptoms such as reduced activity and erect hair. With the prolongation of OVA exposure time, the body weight of mice in the model group was lower than that in the control group, and the difference was statistically significant at the end of the experiment (19.90 ± 1.06 g vs. 20.08 ± 0.87 g, *t* = −0.669, *p* = 0.506) for baseline; 20.66 ± 1.29 g vs. 20.17 ± 1.39 g, *t* = 1.011, *p* = 0.318 for D21; 20.72 ± 1.53 g vs. 21.11 ± 1.29 g, *t* = −0.682, *p* = 0.501 for D28 and 19.89 ± 1.68 g vs. 22.06 ± 1.76 g, *t* = −2.815, *p* = 0.011 for D35 respectively) (Figure 4).

Although feeding lasted for as long as 35 days, the body weight in the model group did not increase with the prolongation of feeding time (19.89 ± 1.68 g at the endpoint vs. 19.90 ± 1.06 g at the baseline, *t* = −0.018, *p* = 0.986).

### 3.2. LESP of Mice

**Figure 5:**
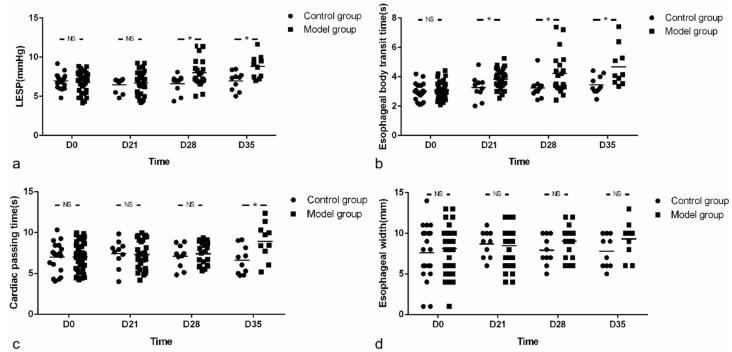
The LESP during baseline and different time points, esophageal emptying test and esophageal radiography. (**a**) Although there was no difference between model group and control group at baseline, the LESP was higher at D28 and D35. (**b**) During OVA treatment, the esophageal body transit time of mice in model group was longer than that of control group at D21, D28 and D35, respectively. (**c**) Although there was no difference between groups at baseline, the cardiac passing time of model group was longer than that of control group at the end of the experiment. (**d**) At every time point, there was no significant difference in the esophageal width between groups. The number of mice in control group was 20, 10, 10 and 10, for each time point (D0-D35). In contrast, the number of mice in model group was 40, 30, 20 and10 for each time point, respectively. Unpaired *t*-tests were used in comparison between groups and among different viewpoints in model group. LESP: lower esophageal sphincter pressure. * *p* < 0.05, NS—not significant.

LESP is an important index for evaluating motility of LES, denoting the resistance of LES to food during swallowing. Increased LESP suggests characteristic esophageal motility disorder in mouse model for AC [19]. Therefore, we also evaluated the LESP of mice and found no differences at baseline (6.80 ± 1.23 vs. 6.96 ± 0.94 mmHg, *t* = −0.507, *p* = 0.614) and D21 (6.71 ± 1.50 vs. 6.47 ± 0.90 mmHg, *t* = 0.462, *p* = 0.647), while the LESP of the model group on D28 (8.01 ± 1.79 vs. 6.63 ± 1.18 mmHg, *t* = 2.191, *p* = 0.037) and D35 (8.81 ± 1.49 vs. 6.98 ± 1.19 mmHg, *t* = 3.020, *p* = 0.007) was higher than that of the control group (Figure 5a).

In addition, in the model group, compared with baseline, LESP increased with the prolongation of OVA treatment and the difference was significant at D28 (8.01 ± 1.79 vs. 6.80 ± 1.23 mmHg, *t* = 3.072, *p* = 0.003) and D35 (8.81 ± 1.49 vs. 6.80 ± 1.23 mmHg, *t* = 4.433, *p* = 0.000).

### 3.3. Esophageal Emptying and Esophageal Radiography

In order to intuitively reflect esophageal emptying, we introduced a novel esophageal emptying test in mice in the present study.

The esophageal body transit time of mice was not different between groups at baseline (3.09 ± 0.53 vs. 3.01 ± 0.59 s, *t* = 0.487, *p* = 0.628), and it was longer in model group than that in control group at D21 (3.82 ± 0.67 vs. 3.27 ± 0.80 s, *t* = 2.140, *p* = 0.039), D28 (4.23 ± 1.40 vs. 3.25 ± 0.75 s, *t* = 2.065, *p* = 0.048) and D35 (4.68 ± 1.35 vs. 3.45 ± 0.62 s, *t* = 2.619, *p* = 0.022) (Figure 5b). Additionally, in the model group, the esophageal body transit time prolonged significantly with the prolongation of OVA treatment compared to that of the baseline (D21 (3.82 ± 0.67 vs.3.09 ± 0.53 s, *t* = 5.122, *p* = 0.000), D28 (4.23 ± 1.40 vs. 3.09 ± 0.53 s, *t* = 3.539, *p* = 0.002) and D35 (4.68 ± 1.35 vs. 3.09 ± 0.53 s, *t* = 3.676, *p* = 0.004), respectively).

The cardiac passing time of mice in the model group was longer than that of the control group at D35 (8.95 ± 2.26 vs. 6.64 ± 1.71 s, *t* = 2.574, *p* = 0.019), while there was no difference in cardiac passing time between groups at the other three time points (7.02 ± 1.71 vs. 7.00 ± 1.78 s, *t* = 0.033, *p* = 0.974 for baseline, 7.31 ± 1.70 vs. 7.44 ± 1.68 s, *t* = −0.208, *p* = 0.836 for D21 and 7.40 ± 1.38 vs. 7.10 ± 1.43 s, *t* = 0.557, *p* = 0.582 for D28, respectively) (Figure 5c). In the model group, with the prolongation of OVA exposure time, the cardiac passing time increased accordingly, and the difference was significant at the end time point (8.95 ± 2.26 vs. 7.02 ± 1.71 s, *t* = 2.998, *p* = 0.004).

There was no esophageal stenosis in both groups under traditional esophageal radiography. The distal esophagus of mice in the model group showed mild beak sign. No significant difference was found in esophageal width between groups at any time points (8.10 ± 2.73 vs. 7.60 ± 3.44 mm, *t* = 0.613, *p* = 0.542 for baseline, 8.47 ± 2.32 vs. 8.70 ± 1.64 mm, *t* = −0.294, *p* = 0.770 for D21; 9.05 ± 1.85 vs. 7.90 ± 1.79 mm, *t* = 1.622, *p* = 0.116 for D28 and 9.30 ± 2.16 vs. 7.80 ± 1.99 mm, *t* = 1.614, *p* = 0.124 for D35, respectively) (Figure 5d). In addition, even though presenting with an increasing trend, the esophageal width of model group did not increase significantly with the prolongation of OVA treatment (9.30 ± 2.16 vs. 8.10 ± 2.73 mm, *t* = 1.291, *p* = 0.203 for D35), suggesting that the esophageal width is not sufficiently sensitive to reflect the esophageal motility in mice.

### 3.4. Esophageal Histopathology of Mice

**Figure 6:**
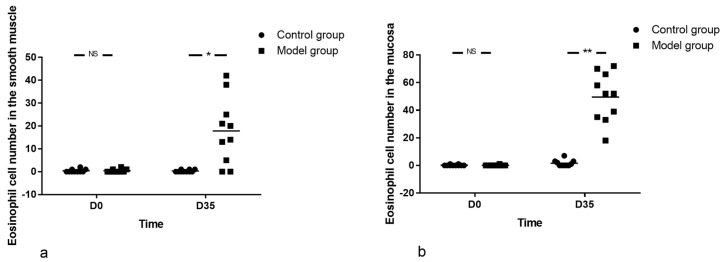
Esophageal histopathology of mice. (**a**) Eosinophil cell number in the smooth muscle and (**b**) Eosinophil cell number in the mucosa. There were almost no eosinophils in the esophagus at baseline in both groups by HE staining. The eosinophils in model group outnumbered that in control group with the prolongation of OVA treatment, in both mucosa and smooth muscle layer of esophagus. The number of mice in both groups was 10 at each time point. Mann-Whitney U-Test was used in comparison between groups and among different viewpoints in model group. * *p* < 0.05, ** *p* < 0.001, NS—not significant.

There was almost no eosinophil in the muscle layer of esophagus in both groups at baseline (0.40 ± 0.70 vs. 0.60 ± 0.84, Z = −0.536, *p* = 0.684). At D35, mice in the model group presented with a larger amount of eosinophil in the muscle layer of esophagus compared with that of control group (17.80 ± 14.51 vs. 0.40 ± 0.52, Z = −2.823, *p* = 0.005) (Figure 6a).

At baseline, there was almost no eosinophil at mucosa of esophagus (0.10 ± 0.32 vs. 0.20 ± 0.42, Z = −0.610, *p* = 0.739). At D35, the eosinophil outnumbered that of control group (49.50 ± 17.85 vs. 1.60 ± 2.27, Z = −3.811, *p* = 0.000) (Figure 6b).

**Figure 7:**
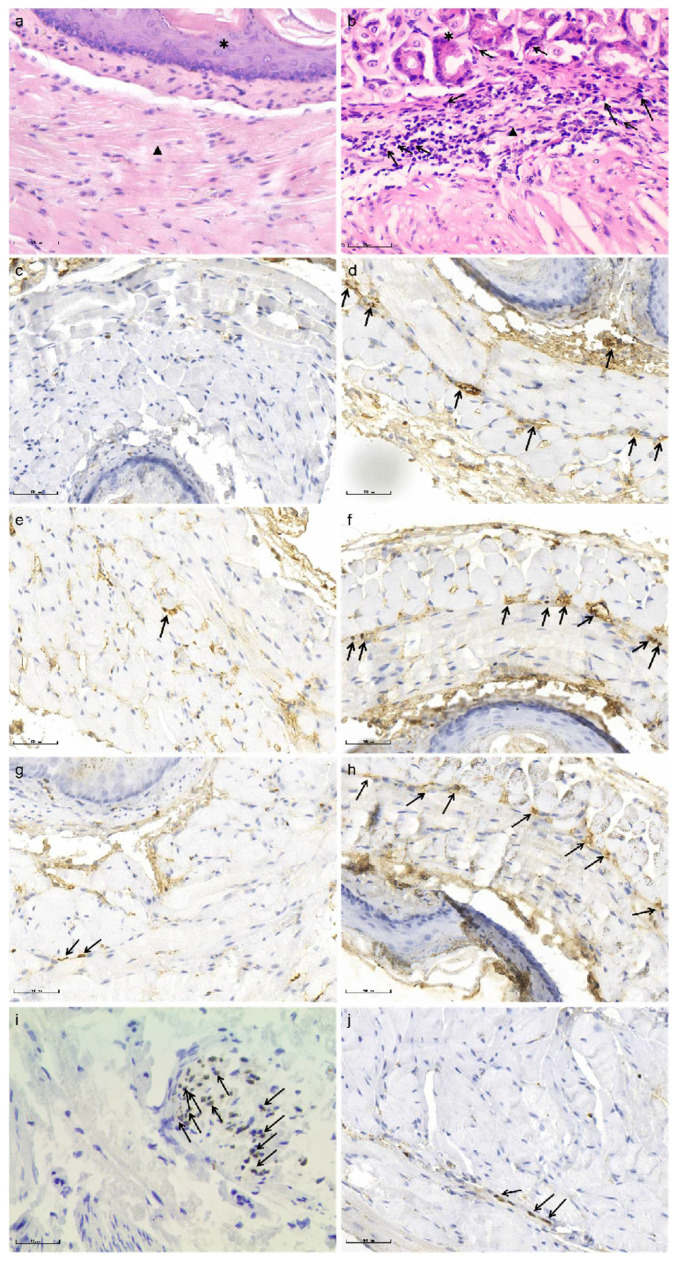
Immuno-histochemical staining of the esophageal tissue of mice in control and model group at D35. (**a**,**b**) HE staining of the esophagus, which shows eosinophils in model group (**b**) outnumbered that in control group (**a**) in both mucosa and muscularis propria. * Indicates mucosa, ▲ indicates muscularis propria, black arrows indicate eosinophils. (**c**–**h**) Immuno-histochemical staining showed more MBP, ECP and EDN positive cells in the esophageal smooth muscle of mice in model group (**d**,**f**,**h**) compared with controls (**c**,**e**,**g**). Black arrows indicate MBP, ECP and EDN positive cells, respectively. (**i**–**j**) Immuno-histochemical staining for the localization of the primary antibody to SOX-10, which indicates ganglion cells in esophageal tissue of mice in model group (**j**) and control group (**i**). Black arrows indicate SOX-10 positive cells. Magnification ×400.

In addition, in the model group, with the prolongation of OVA treatment, eosinophils in the muscle wall of the esophagus increased compared with the baseline (3.90 ± 3.21 vs. 0.40 ± 0.70, *Z* = −2.780, *p* = 0.007 for D28 and 17.80 ± 14.51 vs. 0.40 ± 0.70, *Z* = −2.934, *p* = 0.004 for D35). Likewise, the trend was also prominent in the mucosal layer (6.30 ± 3.74 vs. 0.10 ± 0.32, *Z* = −3.600, *p* = 0.000 for D21, 13.10 ± 8.89 vs. 0.10 ± 0.32, *Z* = −3.596, *p* = 0.000 and 49.50 ± 17.85 vs. 0.10 ± 0.32, *Z* = −3.964, *p* = 0.000 for D28 and D35, respectively) (Figure 6 and Figure 7).

To further verify whether the infiltrated eosinophil was active, we performed immuno-histochemical staining, and found that MBP-positive eosinophils were scattered or focally distributed in the esophageal muscularis propria from the model group, accompanied with scattered ECP and EDN-positive particles at D35. In contrast, no expression of MBP, ECP and EDN was observed in control group (Figure 7).

**Figure 8:**
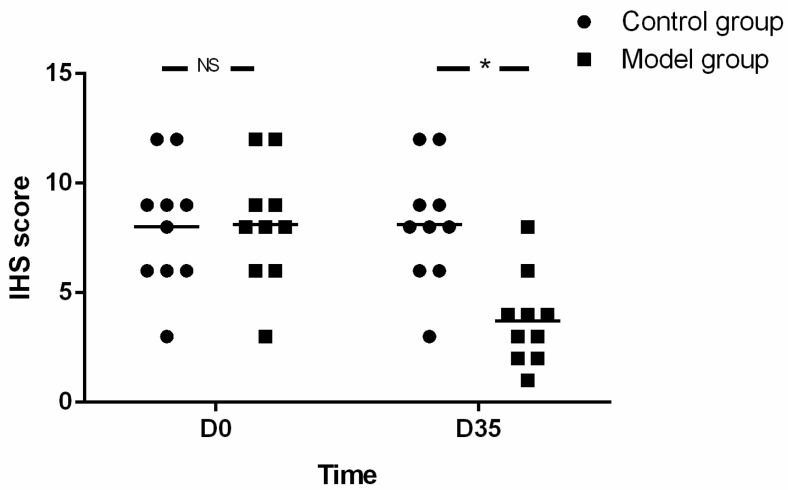
The expression of SOX-10. The expression of SOX-10 in model group was significantly reduced compared with that of control group at the end stage of the experiment. The number of mice in both groups was 10 at each time point. Unpaired two-tailed *t*-Test was used in comparison between groups and among different viewpoints in model group. IHS: immune-histochemical score. * *p* < 0.05, NS—not significant.

Additionally, at baseline, the HIS for SOX-10 was not different between both groups (8.10 ± 2.73 vs. 8.00 ± 2.83, *t* = 0.080, *p* = 0.937), while, at D35, the expression of SOX-10 in mice of model group decreased significantly compared with that of control group (3.70 ± 2.06 vs. 8.10 ± 2.73, *t* = −4.074, *p* = 0.001) (Figure 8).

**Figure 9:**
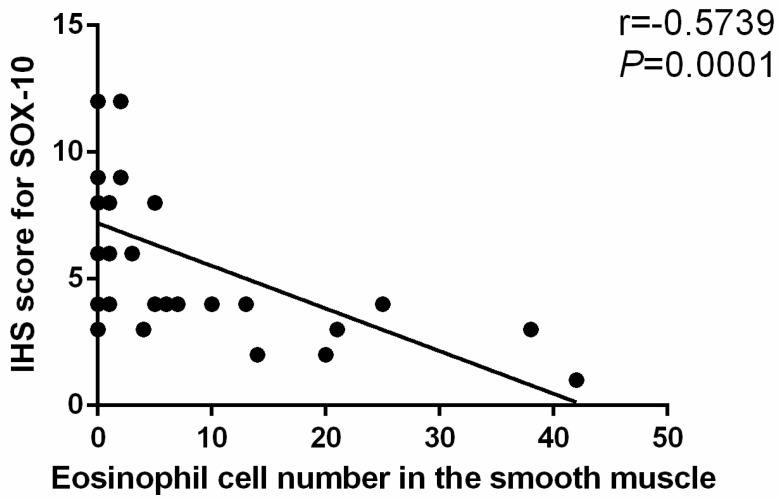
The correlation between eosinophil count and the SOX-10-IR Cells. The number of eosinophils in the smooth muscle layer of esophagus was negatively correlated with SOX-10-IR Cells. n = 40, r = −0.5739, r^2^ = 0.3294, *p* = 0.0001, 95% CI (−0.7512, −0.3195). IHS: immuno-histochemical score. Spearman rank correlation was used.

Contrary to the gradual increase in the number of eosinophils, the expression of SOX-10 decreased over time with the prolongation of OVA treatment (5.20 ± 1.87 vs. 8.10 ± 2.73, *t* = −2.772, *p* = 0.013 for D28 and 3.70 ± 2.06 vs. 8.10 ± 2.73, *t* = −0.563, *p* = 0.001 for D35 respectively). There was also a negative correlation between the eosinophil count and the SOX-10-IR cells in the muscle layer of esophagus (r = −0.5739, *p* = 0.0001, 95% CI (−0.7512, −0.3195)) (Figure 9).

## 4. Discussion

Herein, mice were sensitized by OVA for as long as 35 days. At the end of the study, eosinophil infiltration in muscularis propria and AC-like esophageal dismotility were observed. In addition, eosinophil count was negatively correlated with SOX-10-IR cells. As no esophageal stenosis was found by X-ray esophageal fluoroscopy, it is logical to speculate that eosinophil infiltration in the muscle layer of the esophagus may induce the esophageal dismotility and loss of SOX-10-IR cells, which may be related to the release of eosinophil secretion products with muscle and nerve activities, disturbance of peristalsis and LES relaxation, or release of cytotoxic eosinophil secretion products, such as ECP and EDN [8]. As expected, in this study, ECP and EDN were both identified in the esophageal muscle wall, which supported the speculation. To the best of our knowledge, there is synchronized decrease of neurons and glial cells in esophagus of AC [20], therefore we speculated that the loss of SOX-10-IR cells (positive for both neurons and glial cells), can indicate the damage of esophageal myenteric plexus in this study. Obviously, the results were preliminary and need verification by specific antibodies against neurons and glial cells, and the effects of inflammation on neurons and glial cells of esophagus need further exploration in the future.

In addition to eosinophil, OVA can also induce mast cell infiltration in the esophagus wall [21]. Mavi found that the esophageal motility dysfunction was dependent on mast cell inflammation in a trans-genetic EoE murine model [22]. In a previous study, only muscle strips were used to reflect the motility of the esophagus in vitro. Since the esophageal movement is complex and highly coordinated, data from muscle strips cannot be used alone to reflect the motility of the esophagus in vivo. More importantly, in previous study, the extent of eosinophils and mast cells infiltration in esophageal muscle layer and the count of myenteric neurons were not clarified. Therefore, the previous study cannot exclude the possibility that eosinophil infiltration leads to the esophageal dysmotility.

Both eosinophil and mast cell can be found in the specimens from muscularis propria of AC patients [5,7,23]. In addition to the inconsistent evidence listed above, anecdotal clinical evidence has even suggested that administration of prednisolone on AC results in a complete disappearance of dysphagia because of improved esophageal motility and reduced eosinophilic infiltrate [24], which makes the situation more confusing. It is still not clear which inflammatory cells contribute the most to the occurrence of AC to date. On this issue, this OVA driven mouse model with AC-like esophageal histopathological and functional characteristics provides a suitable platform for future research.

The depth of eosinophil infiltration into the esophagus is related to types of respiratory antigen and the exposure procedures. Simple respiratory allergenic (such as dog, cat, cockroach, dust mite and A. fumigatus) stimulation, lasting for 21–28 days, could promote the infiltration of eosinophil, mainly in the epithelium [12,25]. However, if the mice were sensitized by intraperitoneal injection of OVA and then exposed to OVA via respiratory tract for 24 days, the eosinophil infiltrated more deeply, with most of the eosinophils infiltrated into the mucosa and submucosa [15]. In support of previous findings, we also observed the eosinophils infiltration in esophageal mucosa and submucosa from D21. As expected, the eosinophil infiltrated in muscularis propria at the end of OVA treatment, suggesting the prolonged OVA treatment was conducive to the deepening of eosinophil infiltration in the esophageal wall. This may be due to the first sensitization promotes specific T and B cell responses and the subsequent allergic developments [26]. However, the mechanism still needs to be verified in the future.

Regarding the number of eosinophils, it was reported that the eosinophil count in the esophagus fluctuated with the extension of antigens exposure. For cockroach (applied to the nares), the eosinophil count in esophageal wall increased in an ‘S’ shape, peaked at the second week after intervention, then decreased, but it continued to rise from the third week, and reached the maximum at the fourth week. For dust mite (applied to the nares), the change of eosinophil count in esophageal wall showed an inverted ‘V’-shaped curve, peaking at the third week [25]. Likewise, in IL-13 transgenic mice with esophageal eosinophilia, the eosinophil count in the esophagus peaked at day 10 (dietary doxycycline-impregnated food exposure lasted for a total of 30 days), showing an inverted ‘V’ shape [27]. We were also interested in the kinetics of eosinophilia in the esophagus and found the number of eosinophils in the esophageal wall increased with the prolongation of OVA exposure time. However, contrary to previous studies, the increase curve was not ‘S’-shaped or inverted ‘V’ shaped but linear in the present study. This may be related to the different schemes adopted or to the possibility that treatment did not last long enough to reach the peak of eosinophil infiltration. In order to clarify the kinetics of eosinophil infiltration, the OVA treatment time should be prolonged and the observation period of withdrawal should also be included in the future.

The emptying test, as an intuitive and accurate method to evaluate gastrointestinal motility, is very useful in clinical work, especially in evaluating the therapeutic effect of AC [28,29]. However, it is difficult to perform in mouse because the amount of barium in the esophagus cannot be accurately determined over time in vivo, and more importantly, mouse cannot cooperate with this examination. Since radiography can only show indirect information on the motility of esophagus, we introduced a novel esophageal emptying test in this study. As a result, in contrast to the almost unchanged esophageal width, the emptying test reflected the movement of esophagus more sensitively and the emptying time is easy to calculate by replacing barium meal with a tiny metal ball that is easy to locate. Therefore, this method deserves to be further validated in the future.

Of course, our study has limitations. Firstly, although the eosinophil infiltration in esophageal muscle layer was prominent, the possibility of other inflammatory cells infiltration at the same site could not be excluded. Secondly, the infiltration of eosinophils in other parts of the digestive tract was not observed in the present study, so it cannot reflect the overall picture of the impact of long-term antigen exposure on the digestive tract. Thirdly, the mice can only reflect characteristics of a subgroup of AC with eosinophil in the muscle layer of the esophagus. We must acknowledge, since the method was based on an EoE model, that the infiltration of eosinophil in mucosa of esophagus is unavoidable. Finally, based on the EoE model sensitized by respiratory antigens, this study did not compare the esophageal pathology and motility of other sensitization methods. Nonetheless, this was the only study specifically devoted to explore the eosinophil infiltration in muscularis propria as well as its relationship with esophageal motility in mice.

Taken together, this study suggests that long-term OVA treatment, assisted by alum, may induce eosinophil infiltration in the esophageal muscularis propria and AC-like dysmotility of the esophagus. Although further verification is still needed, the method discussed in the present study is helpful to explore the effect of eosinophil infiltration in the pathogenesis of some AC patients, and even to formulate corresponding treatment plans in the future. Interestingly, it also prompts us to doubt whether respiratory antigen can induce esophageal dysmotility by promoting eosinophil infiltration in esophageal muscularis propria.

## Data Availability

The data presented in this study are available on request from the corresponding author.
